# Anopheline and culicine mosquitoes are not repelled by surfaces treated with the entomopathogenic fungi *Metarhizium anisopliae *and *Beauveria bassiana*

**DOI:** 10.1186/1756-3305-3-80

**Published:** 2010-08-27

**Authors:** Ladslaus L Mnyone, Constantianus JM Koenraadt, Issa N Lyimo, Monica W Mpingwa, Willem Takken, Tanya L Russell

**Affiliations:** 1 Biomedical and Environmental Group, Ifakara Health Institute, P.O. Box 53, Off Mlabani Passage, Ifakara, Tanzania; 2 Laboratory of Entomology, Wageningen University and Research Centre, P.O. Box 8031, 6700 EH, Wageningen, The Netherlands; 3 Pest Management Centre, Sokoine University of Agriculture, P.O. Box 3110, Morogoro, Tanzania; 4 Faculty of Biomedical and Life Sciences, University of Glasgow, 120 University Place, G12 8TA, Glasgow, UK; 5 Vector Group, Liverpool School of Tropical Medicine, Liverpool, L3 5QA, UK; 6 The University of Queensland, School of Population Health, Australian Centre for Tropical and International Health, Brisbane, 4006, Australia

## Abstract

**Background:**

Entomopathogenic fungi, *Metarhizium anisopliae *and *Beauveria bassiana*, are promising bio-pesticides for application against adult malaria mosquito vectors. An understanding of the behavioural responses of mosquitoes towards these fungi is necessary to guide development of fungi beyond the 'proof of concept' stage and to design suitable intervention tools.

**Methods:**

Here we tested whether oil-formulations of the two fungi could be detected and avoided by adult *Anopheles gambiae s.s., Anopheles arabiensis *and *Culex quinquefasciatus*. The bioassays used a glass chamber divided into three compartments (each 250 × 250 × 250 mm): release, middle and stimulus compartments. Netting with or without fungus was fitted in front of the stimulus compartment. Mosquitoes were released and the proportion that entered the stimulus compartment was determined and compared between treatments. Treatments were untreated netting (control 1), netting with mineral oil (control 2) and fungal conidia formulated in mineral oil evaluated at three different dosages (2 × 10^10^, 4 × 10^10 ^and 8 × 10^10 ^conidia m^-2^).

**Results:**

Neither fungal strain was repellent as the mean proportion of mosquitoes collected in the stimulus compartment did not differ between experiments with surfaces treated with and without fungus regardless of the fungal isolate and mosquito species tested.

**Conclusion:**

Our results indicate that mineral-oil formulations of *M. anisopliae *and *B. bassiana *were not repellent against the mosquito species tested. Therefore, both fungi are suitable candidates for the further development of tools that aim to control host-seeking or resting mosquitoes using entomopathogenic fungi.

## Introduction

Laboratory [1-3] and small scale field trials [4,5] have demonstrated that malaria vectors can succumb to entomopathogenic fungus infection. Furthermore, these fungi can equally infect and kill insecticide-resistant and insecticide-susceptible malaria vectors [6-8]. In these views, entomopathogenic fungi are increasingly attracting attention as potential biological control agents against malaria vectors, particularly as they are considered to be evolutionary proof agents, against which resistance is less likely to develop [9]. As such, entomopathogenic fungi have the potential to be used as a chemical insecticide resistance management tool. Fungal infection restored part of the insecticide susceptibility of kdr-resistant anopheline mosquitoes [8] suggesting that fungal infections may extend the lifetime of insecticidal control strategies.

For fungal infection to occur, the conidia need to contact the host, after which they attach to, germinate, and penetrate the cuticle [10]. Once within the host mosquito, the hyphae proliferate whilst exploiting nutritional resources and release toxic metabolites that eventually cause sub-lethal and lethal effects to the host [11]. Normally, the host dies from a combination of mechanical damage, nutrient depletion and toxicosis. Mechanical damage causes loss of host's cell membrane integrity and fluids, which in turn leads to decomposition of internal organs [11]. Examples of sub-lethal effects are reduced blood feeding propensity and fecundity of the mosquitoes [12].

Success or failure of infection depends on the nature of host-parasite interaction, which can be altered by physiological, ecological and behavioural conditions of the host [13]. Many studies on host-pathogen interaction in arthropods have focused on physiological and ecological alterations, with little attention paid to behavioural alterations. One of the most important behaviours is the host insect's ability to detect and avoid fungal conidia. Termites [13-15], ants [13] and groundnut beetles [16] were all shown to detect and avoid *Metarhizium anisopliae*. Behavioural avoidance was also observed in adults of the common flower bug (*Anthocoris nemorum*) [17] and ladybirds (*Coccinella septempunctata*) that could both detect and avoid *B. bassiana *conidia [18]. In the field of mosquito control, if conidia can repel mosquitoes this could minimize mosquito contact with conidia, and thus reduce the efficacy of this control tool. Entomopathogenic fungi that are either non-repellent or attractive would be more desirable unless their ability to repel is strong enough to prevent mosquitoes from entering human houses and biting people. Laboratory studies to optimize fungal formulations of *M. anisopliae *and *B. bassiana *have been conducted [3] and provide a foundation for conducting field-based efficacy studies. Understanding how mosquitoes respond behaviourally to fungal exposure is thus essential. Avoidance behaviour may hamper the efficacy and the overall epidemiological impact of the fungus. We therefore tested the behavioural response of *An. gambiae s.s*., *An. arabiensis *and *Culex quinquefasciatus *after contacting or detecting conidia of *M. anisopliae *and *B. bassiana. Culex quinquefasciatus *are susceptible to entomopathogenic fungi and under field settings they often appear together with malaria vectors, thus both may be targeted. Most importantly, *Culex quinquefasciatus *cause nuisance and are important vectors of filariasis. Therefore, understanding how they respond to the fungus was also deemed important since targeting both vectors would be more cost-effective and possibly enhance societal adoption of the technology. Behavioural responses can vary with conidia dose [19]; therefore, we tested different conidia doses formulated in pure mineral oil.

## Materials and methods

### Mosquitoes

Mosquitoes used in this study were obtained from insectary colonies maintained in the Ifakara Health Institute (IHI), Tanzania. The *An. gambiae s.s*. colony was established from a population near Njage village, Tanzania, in 1996. The *An. arabiensis *colony was established from Sagamaganga village, Tanzania in 2007. Larvae and adults were reared using procedures described by Huho et al [20]. The *Cx. quinquefasciatus *colony was established from Ifakara village, Tanzania in 2009; using similar procedures as with anophelines except that adults were blood-fed on pigeons. The study was performed using 3-7 d old unfed adult female mosquitoes that were starved at least 6 h before use.

### Fungal isolates, formulation and application

Two fungal isolates were used: 1) *M. anisopliae var. anisopliae *ICIPE-30, isolated in 1989 from the maize stalk borer, *Busseola fusca *(Lepidoptera, Noctuidae) in Western Kenya, and 2) *B. bassiana *I93-825 (IMI 391510), isolated from a chrysomelid beetle (Coleoptera) in the USA. Dry conidia of *M. anisopliae *were produced at IHI, after passaging and re-isolation from infected mosquito cadavers. Conidia were harvested from 15 d old cultures grown on rice grains. Dry conidia of *B. bassiana *were imported from Penn State University, USA (courtesy M.B. Thomas, Penn State University, USA). Conidia were formulated in highly refined mineral oil, Enerpar (Enerpar M002^®^, BP Southern Africa Ltd). Preparation and application of fungal formulations was done using procedures described by Mnyone et al [3]. After the treatment of exposure netting (conidia formulation or oil) it was left to dry for 24 h at ambient conditions.

### Behaviour chamber

**Figure 1:**
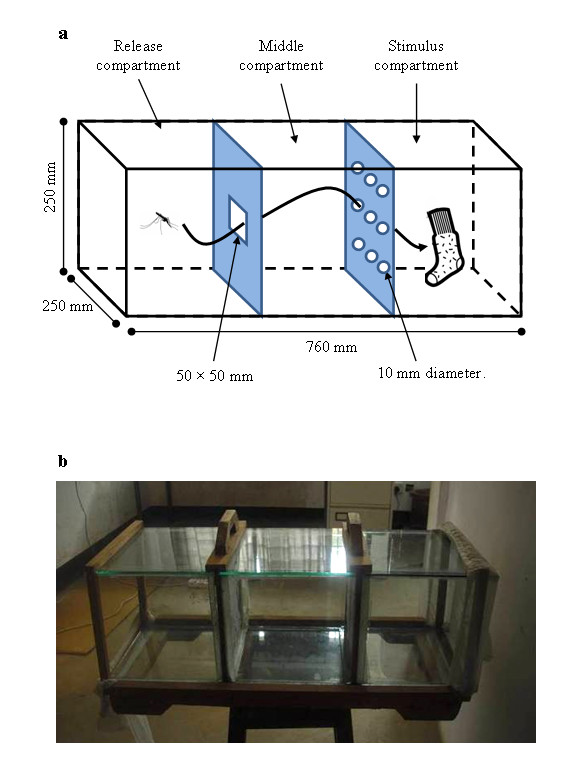
**Behavioural chamber with three equally sized compartments: release, middle, and stimulus compartment**. A guinea pig (Experiment 1) or a worn sock (Experiment 2) was placed in the stimulus compartment.

A glass chamber with three equally sized compartments (250 × 250 × 250 mm) was used: release, middle and stimulus compartments (Figure 1). The release compartment was separated from the middle compartment by a plywood frame fitted with white paper with a square opening at the middle (50 × 50 mm) to allow mosquitoes to move into the adjacent compartment. The middle and stimulus compartments were separated by a plywood frame fitted with a piece of polyester netting. The netting contained three rows of circular holes (10 mm diameter) with each row containing three holes. The distance between holes within and between adjacent rows was 50 mm. The ends of the glass chamber were covered with a transparent piece of cloth to prevent mosquitoes from escaping. The different treatments (three concentrations of fungal conidia suspended in mineral oil, mineral oil only and untreated) were applied to the netting separating the middle from stimulus compartments. In each replicate, freshly treated netting was used. To attract mosquitoes into the stimulus compartment, via the exposure netting, a host odour in the form of a guinea pig or worn sock was placed into the stimulus compartment. When used, the guinea pig was restrained within a plywood box covered with netting to protect guinea pigs from mosquito bites. The glass chamber was cleaned with distilled water and 70% alcohol in between trials and left to dry in open air to prevent transferring residual effects to the subsequent trials. Four glass chambers were used in parallel, corresponding to the four different treatments as detailed in the experimental procedures below. Air flow inside the experimental room was passive.

## Experimental procedures

### Experiment 1

Two doses (2 × 10^10 ^and 4 × 10^10 ^conidia m^-2^) of *M. anisopliae *and *B. bassiana *were tested against *An. gambiae s.s*. and *An. arabiensis *and compared with oil only and untreated controls. The two treatments and controls were run concurrently. Fungal isolates were tested each at a time; and species of mosquitoes were tested against a particular fungal isolate each at a time. About 70 mosquitoes were placed into the release compartment in the early evening (6:00 pm) and were free to move towards the stimulus compartment overnight. The stimulus compartment for treatments and controls consisted of a guinea pig. The next morning (7:00 am), mosquitoes were collected from each compartment, killed and counted. The trial was repeated four times, each time with fresh mosquitoes, to obtain four replicates for each experimental factor.

### Experiment 2

The set up and procedures were similar as described for Experiment 1, however, with the following exceptions. The experiment tested only *B. bassiana *against *An. gambiae s.s*., *An. arabiensis *as well as *Cx. quinquefasciatus*. The conidial concentrations tested were: 2 × 10^10 ^and 8 × 10^10 ^conidia m^-2^. The stimulus compartment for treatments and controls consisted of socks worn by a human volunteer [21]. One sock was used per each glass chamber. The socks were worn for 12 h; and used immediately after being put off. Each trial was repeated six times to obtain six independent replicates. The treatments and controls were run concurrently.

### Data analysis

The proportion of the mosquitoes released that were collected in the stimulus compartment was the output measure; it was calculated as a ratio of the number in stimulus compartment to the total number of mosquitoes (number in release, middle and stimulus compartments). Data were arcsine transformed to meet the assumption of standard normal distribution; then analysis of variance (ANOVA) was performed to compare different treatments. SPSS version 17 was used.

## Results

### Experiment 1

**Figure 2:**
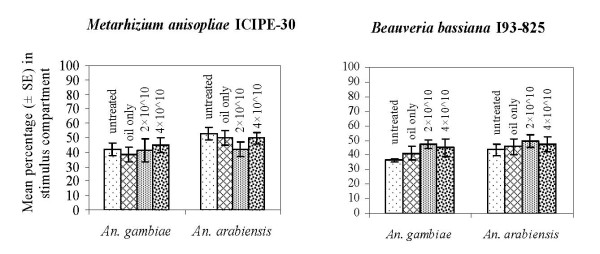
**Proportions (Mean ± SE) of *Anopheles gambiae s.s*. and *Anopheles arabiensis *mosquitoes collected in the stimulus compartment with untreated control, mineral oil only control, and two formulations of *Metarhizium anisopliae *ICIPE-30 and *Beauveria bassiana *I93-825**.

For *M. anisopliae*, the mean proportions (± SE) of *An. gambiae *that entered the stimulus (guinea pig) compartment were: untreated control 42.1 ± 4.2%, mineral oil only 37.9 ± 5%, conidia dose 2 × 10^10 ^41 ± 8.1% and conidia dose 4 × 10^10 ^44.9 ± 5%. This difference was not statistically significant (F = 0.24; df = 3,12; *p *= 0.87; Figure 2). The mean proportions for *An. arabiensis *were: untreated control 52.8 ± 4.3%, oil only 49.9 ± 5.2%, conidia dose 2 × 10^10 ^41.6 ± 5.2% and conidia dose 4 × 10^10 ^49.6 ± 4.2%. This difference was also not statistically significant (F = 1.0; df = 3,12; *p *= 0.43, Figure 2). For *B. bassiana*, mean proportions of *An. gambiae *that entered the stimulus chamber were: untreated control 36.4 ± 1.2%, mineral oil only treated netting 40.9 ± 4.6%, conidia dose 2 × 10^10 ^47.5 ± 2.8% and conidia dose 4 × 10^10 ^44.8 ± 6.4%. This difference was not statistically significant (F = 1.27; df = 3,12; *p *= 0.33; Figure 2). Mean proportions of *An. arabiensis *were: untreated control 43.48 ± 4.2%, oil only 45.7 ± 5.6%, conidia dose 2 × 10^10 ^49.7 ± 4.4%, and conidia dose 4 × 10^10 ^47.1 ± 5.1%. This difference was also not statistically significant (F = 0.27; df = 3,12; *p *= 0.84, Figure 2).

### Experiment 2

**Figure 3:**
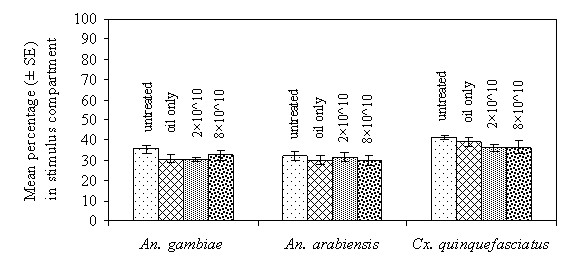
**Proportion (Mean ± SE) of *Anopheles gambiae s.s*., *Anopheles arabiensis *and *Culex quinquefasciatus *collected in the stimulus compartment with untreated control, mineral oil only control, and two formulations of *Beauveria bassiana *I93-825**.

Mean proportions of *An. gambiae *that entered the stimulus (worn sock) compartment were: control 35.4 ± 2.1%, oil-only control 30.7 ± 2%, conidia dose 2 × 10^10 ^30.5 ± 1% and conidia dose 8 × 10^10 ^32.7 ± 2.7%. This difference was not statistically significant (F = 1.19; df = 3,20; *p *= 0.34; Figure 3). Mean proportions for *An. arabiensis *were: untreated control 32.1 ± 2.1%, oil only 30.1 ± 2%, conidial dose 2 × 10^10 ^31.5 ± 2.3% and conidial dose 8 × 10^10 ^30.1 ± 2.3%. This difference was not significant (F = 0.21; df = 3,20; *p *= 0.89). Mean proportions for *Cx. quinquefasciatus *were: untreated control 41.5 ± 1.1%, oil only 39.2 ± 2.4%, conidia dose 2 × 10^10 ^36.1 ± 1.7% and conidia dose 8 × 10^10 ^36.4 ± 3.2%. The difference was also not statistically significant (F = 1.37; df = 3,20; *p *= 0.28: Figure 3).

## Discussion

Successful fungal infection depends on the host contacting treated surface and receiving a threshold dose of infective conidia [22,23]. Results of our two experimental bioassays indicated no repellency of conidia against the three mosquito species tested: Similar proportions of mosquitoes traversed the netting with and without fungus into the stimulus compartment. Scholte et al [24] observed a moderate repelling effect of *M. anisopliae *dry conidia on *An. gambiae s.s*. The repelling effect, however, disappeared after the conidia were suspended in vegetable oil. In our study, although a different type of oil was used (mineral oil, Enerpar), the oil might have similarly suppressed the moderate repelling effect of the conidia. In a field study in Tanzania, a large proportion of wild anophelines was found sitting on fungus-impregnated sheet [4]. Possibly, the oil film prevents conidia from free dispersion in the air and thus reduces the probability of flying mosquitoes encountering conidia [24] or masks the conidia odour. Interestingly, there are several other benefits gained from formulating conidia in oils. Conidia are more efficacious when formulated in oil than water [25]. Compared to water, oil as carrier offers better adhesion and spreading of the formulation on the lipophilic insect cuticle. Furthermore, the oil can form a film on the host cuticle that acts as a humectant, creating good conditions for conidia to germinate and invade the host [25]. Mineral oil can also improve the tolerance of conidia to extreme temperatures.

The behavioural responses of the arthropod hosts to the fungus may vary with the species of fungus, its virulence and conidia concentration. For example, termites, *Coptotermes formosanus*, are able to discriminate the species of fungi by their species-specific odors [19]. It was also found that the antennal response increased with increasing concentrations of suspension in the range from 10^3 ^to 10^7 ^conidia ml^-1 ^[19]. As such, species-specific evaluations will need to be undertaken before other fungal species or concentrations can be developed for use against specific disease vectors. Importantly, in the present study none of the two fungal isolates were repellent at three conidia doses tested, which represent dose rates that have been recommended for field use [3].

The absence of a repellent effect of *M. anisopliae *and *B. bassiana *conidia in our experiments could be beneficial in different ways. There is the possibility for infecting mosquitoes by the lure-and-kill principle [26], using for example odour-baited extra-domiciliary targets [5], since the fungal formulations do not have a repellent affect that would interfere with the attraction to lures. Such lack of a repellency of entomopathogenic fungi against target mosquitoes will also enable entomopathogenic fungi to be integrated into use alongside the existing control tools. In a combination strategy with insecticide-treated bed nets (ITNs), mosquitoes deflected due to moderate repellency of synthetic insecticides, could be pushed to alternative surfaces treated with entomopathogenic fungus. In this way, the combined impact of ITNs and entomopathogenic fungi could be synergistic. Theoretical models suggest that when ITNs and fungi are combined the impact on malaria transmission is equivalent to the additive effect of each intervention alone [27].

## Conclusion

Oil-formulations of *M. anisopliae *ICIPE-30 and *B. bassiana *I93-825 were not found to repel *Anopheles gambiae s.s*., *An. arabiensis *and *Culex quinquefasciatus*, thus, emphasizing the potential of using either fungi for the control of vector mosquitoes.

## Competing interests

The authors declare that they have no competing interests.

## Authors' contributions

Conceived and designed the experiments: LLM, TLR, WT. Performed the experiments: LLM, MWM, INL. Analyzed the data: LLM, CJMK, INL. Wrote the paper: LLM, TLR. Reviewed the paper: CJMK, WT.
